# Hazards of Repeat Pregnancy during Adolescence: A Case-control Study

**DOI:** 10.1055/s-0038-1666811

**Published:** 2018-08

**Authors:** Rafael Bessa de Freitas Galvão, Camilla Olivares Figueira, Anderson Borovac-Pinheiro, Daiane Sofia de Morais Paulino, Debora Bicudo Faria-Schützer, Fernanda Garanhani Surita

**Affiliations:** 1 Department of Obstetrics and Gynecology, School of Medical Sciences, Universidade Estadual de Campinas, SP, Brazil

**Keywords:** adolescence, pregnancy, vulnerability, contraception, adolescência, gravidez, vulnerabilidade, contracepção

## Abstract

**Objective** To evaluate the social, obstetric and psychological risk factors related to repeat pregnancy in teenagers.

**Methods** A case control study conducted at Centro de Atenção à Saúde Integral da Mulher (Caism, in the Portuguese acronym), in Campinas, Brazil, from 2015 to 2017. Three groups were selected: a case-group of adolescents who had repeat pregnancy and two control-groups, one consisting of adolescents who had delivered at first time and another one of adult women with more than one deliveries. Participants were asked about habits, socio-demographics characteristics, reproductive and obstetric history and assessed psychological issues.

**Results** Ninety women were enrolled, 30 in each study group. Adolescents with repeat pregnancy have lower self-esteem scores and more ineffective contraceptive use. When compared with teens at first delivery, they had less schooling level (odds ratio [OR] 4.03 [1.37–11.8]), more school abandon (OR 8.16 [2.36–28.2]) and drugs use (OR 4.97[1.39–17.8]). Non-white skin color (OR 6.2 [1.15–41.0]), drugs use (OR 17.5 [2.62–116.6]) and first sexual intercourse under 15y (OR 18.0[2.82–115.0]) were found as higher risk factors for repeat pregnancy when comparing adolescents and adults. Moreover, adolescents with more than one gestation had lower self-esteem and greater susceptibility to unplanned pregnancy.

**Conclusion** There was an association between repeat pregnancy among adolescents and lower education, early onset of sexual activity, non-white skin color, low use of contraception and increased use of drugs.

## Abstract (pt)

**Resumo:**

**Objetivo** Avaliar a associação entre fatores de risco sociais, obstétricos e psicológicos relacionados a repetição da gravidez em adolescentes.

**Métodos** Estudo caso-controle realizado num Hospital Universitário de Campinas, Brasil, de 2015 a 2017. Foram selecionados três grupos: um grupo-caso de adolescentes com repetição de gravidez, e dois grupos controles, um de adolescentes primíparas e outro de mulheres adultas com mais de um parto. Foram coletados dados referentes a aspectos sociodemográficos, história reprodutiva e obstétrica e fatores psicológicos.

**Resultados** Foram incluídas 90 mulheres, 30 em cada grupo de estudo. Adolescentes com repetição de gravidez apresentaram menor autoestima e mais uso inadequado de contraceptivos. Quando comparadas a adolescentes primíparas, tiveram menor nível escolar (razão de probabilidades [RP] 4.03 [1.37–11.8]), maior interrupção dos estudos (RP 16.3 [3.61–73.6]) e maior uso de drogas (RP 4.97[1.39–17.8]). A comparação entre adolescentes com repetição de gestação e mulheres adultas revelou maior risco para cor da pele não-branca (RP 6.2 [1.15–41.0]), uso de drogas (RP 17.5 [2.62–116.6]) e primeira relação sexual com menos de 15 anos 15y (RP 18.0 [2.82–115.0]). Além disso, as adolescentes com segunda gravidez apresentam menor autoestima, baixo uso de contracepção e maior suscetibilidade a gravidez não planejada.

**Conclusão** Houve associação entre repetição de gravidez na adolescência e menor tempo de estudo, início precoce de atividade sexual, cor da pele não branca e maior uso de drogas.

## Introduction

Adolescent pregnancy is a global health problem and can be associated with high rates of maternal death and adverse newborn outcomes. The World Health Organization (WHO) defines adolescents as those between the ages of 10 and 19 years old.^1^ In the United States, it is estimated that 35% of adolescents will have a second pregnancy in less than 2 years, and most of those are unintended.^2^

Teenagers tend to postpone the beginning of prenatal care and need careful attention to detect common conditions in this age group, such as use of alcohol, drugs and smoking, besides the higher risk of sexually transmitted infections.^3^ For these young girls, pregnancy means a higher risk during prenatal care and childbirth, as they are at higher risk of preeclampsia and other hypertensive disorders, like anemia, inadequate nutrition, sexually transmitted diseases, low birth weight, fetal growth restriction and prematurity.^4^ Therefore, the infant mortality rate is higher among children born from adolescent mothers compared with those born from young adult women, aged from 20 to 24 years old.^5^

Adolescent pregnancy constitutes a social problem, as there are social factors involving and determining pregnancy at a young age and the consequences related to childbirth at this age. Childbearing during this period feeds a cycle of deprivation that can compromise young mothers and their children's lives, leading to social disadvantage, with higher rates of unemployment, poverty and discrimination. Up to 40% of adolescent mothers feel depressive and stigmatized by the pregnancy; furthermore, low self-esteem is very common among them.^4^

There are several risk factors that can be associated with teenage pregnancy.^3^ ^6^ Apparently, socioeconomic characteristics are the major risk factor, although some may argue that socioeconomic status is more related to a prognostic evaluation of those women. Some studies have not been able to identify factors that are related to incidence of a second pregnancy during adolescent stage. Therefore, there is no evidence of predictors for a higher risk of repeat pregnancy under age of 20.^5^ Some studies have shown obstetric outcomes as an independent determining factor in the incidence of adolescent repeated pregnancies, observing longer inter-pregnancy intervals among women who experienced prenatal and/or childbirth complications.^7^ However, it has been also observed the opposite, as many women with history of poor outcomes during their first pregnancy tend to have a rapid repeat pregnancy, with a less than 12 months between deliveries interval.^8^

It appears that, to reduce adolescent pregnancy rates in developed and developing countries, actions should be focused on a multifaceted and interdisciplinary management that address not only the risk factors and risk-behavior, but also aims for social and cultural factors that influence young people's decision making.^9^ It means that contraception promotion (especially the use of long-acting reversible contraceptives), and sexual orientation should be considered when counseling adolescents,^10^ but those alone may not be enough to decrease adolescent pregnancy rates. A single component action could not be the solution.

This study aimed to evaluate social, obstetric and psychological risk factors related with repeat pregnancy among adolescents in comparison to those of adolescents after their first delivery and adult women with more than one delivery.

## Methods

This case control study was conducted at Centro de Atenção à Saúde Integral da Mulher (Caism, in the Portuguese acronym), at Universidade Estadual de Campinas (UNICAMP) from August of 2015 to August of 2017.

We considered three groups: a case-group of adolescents (≤ 19 years) who had given birth for the second time, that is, as a result of repeat adolescent pregnancy, hereby referred to as repeat pregnancy teenagers, (RPTeens), and two control groups; the first one composed of teenagers who had given birth for the first time (1^st^PTeens), and the control-group of adult women with repeat pregnancy (adults). For each one of these groups, 30 patients were enrolled as in a convenience sampling.

The women were selected through medical records to check age and parity of the potential participants before the medical interview. The cases and controls that met the inclusion criteria were invited to participate and sign the informed consent form before being enrolled. Women with communication difficulties and any other condition that might lead to misunderstanding the questions were also excluded from the study. Then, a 50-minute interview was conducted, in which the women answered objective questions on sociodemographics, reproductive and obstetric history, habits and comorbidities in a data collection form developed specifically for this study. Psychological issues were assessed using the self-esteem scale of Rosenberg, which rates self-esteem as high, medium or low,^11^ ^12^ ^13^ (low, medium or high self-esteem were considered when the punctuation amounted to < 20, 20–30 or > 30 points respectively) and the evaluation of the participants' relationships with their parents and partners, measured by them using a 1 to 10 grading system. For the questions concerning contraception and intention to get pregnant, the “London Measure of Unplanned Pregnancy” questionnaire, tested and validated by Barrett et al (2004),^1^ was applied.^4^ The questionnaires were filled out with no participant identification. Data were collected during the hospitalization of women 1 to 3 days postpartum in an inpatient unit for mothers and their infants, where a multidisciplinary team operates that prioritizes good mother-child interaction, stimulates breastfeeding and guides care for the newborn.

After data were collected, we compared the groups of adolescents using the Mann-Whitney test for numerical variables, and the Fisher exact test and Chi-squared test were used for categorical variables. For a univariate and multivariate analysis of the RPTeens, we compared this group to the primiparous adolescents using logistic regression, with stepwise selection of variables for the multivariate analysis. The same was done in the comparison with the adult women. The odds ratio was calculated, considering a confidence interval (CI) of 95%.

The Institutional Review Board from the University of Campinas, Brazil, CAAE report 00602612.7.1001.5404, approved this study. All the Strengthening the Reporting of Observational Studies in Epidemiology (STROBE) statement items for a prospective study were followed and checked in this manuscript.^15^

## Results

We collected information of ∼ 90 women, distributed equally between the 3 groups. The median age among 1stPTeens and RPTeens was 16.8 and 18 years, respectively (*p* = 0.003). Among the adults, the median age was 26.5 years.

Table 1 shows the obstetric and sociodemographic characteristics among the participants. Mostly, there were no significant differences between the groups for social characteristics, except for educational achievements and drugs use. Repeat pregnancy during adolescence was associated with lower schooling (36.7% vs 70% of both adults and 1stPTeens; *p* = 0.007 and *p* = 0.019, respectively), higher drop-out rates (80% vs 33.3%) in comparison with 1^st^PTeens (*p* < 0.001) and with adults (*p* = 0.003). The case group (RPTeens) was more often related to drugs use (43.3% vs 13.3%) in comparison with 1^st^PTeens (*p* = 0.010) and adults (*p* < 0.001).

**Table 1:** Comparison between obstetric and sociodemographic characteristics among 1st pregnancy teenagers, repeated pregnancy teenagers and adults (*n* = 90)

Variables	1^st^PTeens n (%)	1^st^PTeens vs RPTeens *p*-value	RPTeens n (%)	RPTeens vs Adults *p*-value	Adults n (%)
Skin color (white)	17 (56.7)	0.197***	12 (40.0)	0.071***	19 (63.3)
Partner (yes)	20 (66,7)	0.067***	26 (86.7)	1.000***	26 (86.7)
No actual schooling	10 (33.3)	< 0.001**	24 (80.0)	0.003**	12 (40.0)
High school or college	21 (70.0)	0.019**	11 (36.7)	0.007**	16 (70.0)
Family income ≥ 3 MW	13 (43.3)	0.598***	11 (36.7)	0.039***	19 (63.3)
Paid work	14 (46.7)	0.510**	10 (33.3)	0.011**	19 (63.3)
Smoking	4 (13.3)	1.000**	5 (16.7)	0.519**	7 (23.3)
Alcohol	8 (26.7)	0.347***	5 (16.7)	0.707***	3 (10.0)
Drugs use	4 (13.3)	0.010***	13 (43.3)	< 0.001***	0 (0.00)
Menarche < 12 years	7 (23.3)	0.390***	10 (33.3)	0.787***	11 (36.7)
First sexual intercourse < 15yrs	18 (60.0)	0.273**	22 (73.3)	< 0.001***	7 (23.3)
First delivery < 15 years	3 (10.0)	0.166***	7 (23.3)	0.011***	0 (0.00)
Prenatal care < 6 visits	1 (3.70)	0.054*	7 (23.3)	0.146*	2 (6.67)
Vaginal delivery	20 (66.7)	1.000***	20 (66.7)	0.787***	19 (63.3)
Birth weight < 2,500kg	5 (16.1)	1.000**	5 (16.1)	0.275**	2 (6.67)
Preterm birth (< 37 weeks)	4 (13.3)	1.000**	5 (16.7)	0.707**	3 (10.0)
Previous contraception (yes)	23 (76.7)	1.000***	(76.7)	0.317***	26 (86.7)

Abbreviations: 1^st^PTeens, group of teenagers at first pregnancy (*n* = 30); Adults, group of adult women with more than one pregnancy (*n* = 30); MW, Brazilian minimum wage; RPTeens, group of non-primiparous teenagers (*n* = 30).

* *p*-value according to Mann-Whitney test.

** *p*-value according to Fisher exact test.

*** *p*-value according to Chi-squared test.

Comparing RPTeens with adults, it was also noted that the case group showed lower frequency of paid work (33.3% vs 63.3% of adults; *p* = 0.039) and lower age of first sexual intercourse, showing sexual activity under the age of 15 more frequently (43.3% vs 13.3% of adults; *p* < 0.001).

Some differences among the groups were confirmed when we made a univariate analysis, which showed that RPTeens presented higher risks for drugs use when compared with 1^st^PTeens (OR 4.97[1.39–17.8]), lower educational level (OR 4.03 [1.37–11.8]) and more school interruption (OR 8.16 [2.36–28.2]). The case group (RPTeens) also seemed to have a higher number of people living in the same home (median of 4.77 vs 3.53; OR 1.39 [1.01–1.90]). These results are shown in Table 2.

**Table 2:** Factors associated with repeat adolescent pregnancy comparing 1st pregnancy teenagers to repeated pregnancy teenagers and adults (Univariate logistic regression = 90)

Variables	Categories	1^st^PTeens n (%)	1^st^PTeens vs RPTeens OR (95%CI)	RPTeens n (%)	RPTeens vs Adults OR (95%CI)	Adults n (%)
Age	Continuous variable (md ± sd)	16.8 ± 1.58	1.90 (1.22–2.95)	18.0 ± 1.10	———-	26.5 ± 3.88
Schooling	High school/college	—	1.00	—	1.00	—
	Less than high school/college	9 (30.0)	4.03 (1.37–11.8)	19 (63.3)	4.03 (1.37–11.8)	9 (30.0)
Actual schooling	Out of school	17 (56.7)	1.00	5 (16.7)	1.00	18 (60.0)
	At school	3 (10.0)	1.13 (0.10–13.4)	1 (3.33)	10.09 (0.36–284.5)	0 (0.00)
	Interrupted school	10 (33.3)	8.16 (2.36–28.2)	24 (80.0)	7.20 (2.15–24.1)	12 (40.0)
Onset of labor	Spontaneous Induced Elective C-section	23 (76.7) 7 (23.3) 0 (00.0)	1.00 1.21 (0.36–4.06) 10.8 (1.01–214.1)	19 (63.3) 7 (23.3) 4 (13.3)	1.00 0.64 (0.20–1.99) 9.00 (0.45–178.7)	19 (63.3) 11 (36.7) 0 (0.00)
N of people at home	Continuous variable (md ± sd)	3.53 ± 1.70	1.39 (1.01–1.90)	4.77 ± 2.43	1.23 (0.89–1.70)	4.13 ± 0.94
Drugs use	No	26 (86.7)	1.00	17 (56.7)	1.00	30 (100)
	Yes	4 (13.3)	4.97 (1.39–17.8)	13 (43.3)	47.0 (2.63–840.9)	0 (0.00)
Family monthly income	≥ 3 salaries	13 (43.3)	1.00	11 (36.7)	1.00	19 (63.3)
	< 3 salaries	17 (56.7)	1.32 (0.47–3.72)	19 (63.3)	2.98 (1.04–8.53)	11 (36.7)
First sexual intercourse	≥15 years	12 (40.0)	1.00	8 (26.7)	1.00	23 (76.7)
	<15 years	18 (60.0)	1.83 (0.62–5.45)	22 (73.3)	9.04 (2.80–29.1)	7 (23.3)
First birth	≥15 years	27 (90.0)	1.00	23 (76.7)	1.00	30 (100)
	<15 years	3 (10.0)	2.74 (0.63–11.8)	7 (23.3)	19.5 (1.06–358.4)	0 (0.00)
Postpartum contraception after second birth	Oral	NE	NE	8 (34.8)	1.00	19 (73.1)
	Injectable			15 (65.2)	7.13 (1.93–26.3)	5 (19.2)
	Others			0 (0,00)	0.46 (0.02–10.6)	2 (7.70)
	No use			7 (23.3)	4.16 (0.95–18.3)	4 (13.3)

Abbreviations: 1^st^PTeens, group of teenagers at first pregnancy (*n* = 30); Adults, group of adult women with more than one pregnancy (*n* = 30); md, median; NA, not applicable; NE, Not evaluated; OR, odds ratio for repeated pregnancy; RPTeens, group of non-primiparous teenagers (*n* = 30); sd, standard deviation.

When we compared the case group with adult women, RPTeens referred lower family income (OR 2.98 [1.04–8.53]). They are more prone to use drugs (OR 47.0 [2.63–840.9]) and had the first sexual intercourse at a younger age, before 15 years old (OR 9.04 [2.80–29.1]). Adolescents in the case group also presented higher risk for lower educational level (OR 4.03 [1.37–11.8]), school abandon (OR 7.20 [2.15–24.1]) and first delivery before the age of 15 (OR 19.5[1.06–358.4]) (Table 2).

The multivariate analysis showed that between 1^st^PTeens and RPTeens, schooling remained as a significantly factor associated with repeat pregnancy (OR 16.3 [3.61–73.6]). In comparison with adults, we found a statistically significant difference for non-white skin color (OR 6.2 [1.15–41.0]), drugs use [OR 17.5 (2.62–116.6)] and first sexual intercourse under 15y (OR 18.0 [2.82–115.0]) as characteristics associated with repeat pregnancy (Table 3).

**Table 3:** Factors associated with repeat adolescent pregnancy comparing (Multivariate logistic regression = 90). 1st pregnancy teenagers to repeated pregnancy teenagers and adults

Variables	Categories	1^st^PTeens n (%)	1^st^PTeens vs RPTeens OR (95%CI)	RPTeens n (%)	RPTeens vs Adults OR (95%CI)	Adults n (%)
Age	Continuous variable (md ± sd)	16.8 ± 1.58	**2.34 (1.39–3.94)**	18.0 ± 1.10		
Actual schooling	Out of school	17 (56.7)	1.00	5 (16.7)		
	At school	3 (10.0)	2.70 (0.14–52.8)	1 (3.33)		
	Interrupted school	10 (33.3)	**16.3 (3.61–73.6)**	24 (80.0)		
Skin color	White			12 (40.0)	1.00	63.3
	Non-white			18 (60.0)	**6.20 (1.15–41.0)**	36.7
Drugs use	No			17 (56.7)	1.00	30 (100)
	Yes			13 (43.3)	**17.5 (2.62–116.6)**	0(0.00)
First sexual intercourse	≥15 years			8 (26.7)	1.00	23 (76.7)
	<15 years			22 (73.3)	**18.0 (2.82–115.0)**	7 (23.3)

Abbreviations: 1^st^PTeens, group of teenagers at first pregnancy (*n* = 30); Adults, group of adult women with more than one pregnancy (*n* = 30); Criterion, stepwise selection of variables; md, median; OR, odds ratio for repeated pregnancy; RPTeens, group of non-primiparous teenagers (*n* = 30); sd, standard deviation; 95%CI, 95% confidence interval.

The evaluation of psychological factors using the self-esteem scale of Rosenberg and the “London Measure of Unplanned Pregnancy” questionnaire showed that RPTeens presented lower self-esteem scores than 1^st^PTeens and Adults (median of 26.8 ± 4.75 vs 31.1 ± 3.12; *p* < 0.001 and 30.9 ± 3.17; *p* < 0.001 respectively). The RPTeens presented significantly more “medium” self-esteem when compared with 1^st^PTeens, who had significantly more “high” self-esteem results (80% of RPTeens vs 47.6% of 1^st^PTeens with self-esteem scores of 20–30 and 16% vs 52.4%, respectively, for scores higher than 30) (Table 4). When we compared RPTeens pregnancy with adults, it was noted that family acceptance of pregnancy was significantly lower among younger women (median score of 9.37 ± 1.33 vs 9.83 ± 0.65; *p* = 0.027). For family relationships, women in the three groups referred relationship with their mothers better than with their fathers and partners however the difference was not significant (data not shown).

**Table 4:** Comparison between 1st pregnancy teenagers, repeated pregnancy teenagers and adults regarding self-esteem scores, intention to become pregnant and contraception before actual pregnancy

Variables	Categories	1^st^PTeens n (%)^a^	1^st^PTeens vs RPTeens (*p*-value)	RPTeens n (%)^b^	RPTeens vs Adults (*p*-value)	Adults n (%)^c^
Self-esteem (md ± sd)	< 20 20–30 > 30	0 (0.00) **10 (47.6)** **11 (52.4)**	**0.018** **	1 (4.00) **20 (80.0)** **4 (16.0)**	0.053**	0 (0.00) 12 (54.5) 10 (45.4)
Intent to be pregnant	Intended Unintended	7 (25.0) 21 (75.0)	0.086***	14 (46.7) 16 (53.3)	0.118***	20 (66.7) 10 (33.3)
Contraception before pregnancy	No use Irregular use Ideal use	**4 (17.4)** **19 (82.6)** 0 (0.00)	**< 0.001** **	**15 (51.7)** **12 (41.4)** 2 (6.90)	**0.002** **	**7 (26.9)** **19 (73.1)** 0 (0.00)

Abbreviations: 1^st^PTeens, group of teenagers at first pregnancy (*n* = 30); Adults, group of adult women with more than one pregnancy (*n* = 30). md, median; RPTeens, group of non-primiparous teenagers (*n* = 30); sd, standard deviation.

** *p*-value according to Fisher exact test.

*** *p*-value according to Chi-squared test.

a Missing data from 9 women for self-esteem score, 2 women for answer about intention to become pregnant and 7 women for answer about contraception use before pregnancy.

b Missing data from 5 women for self-esteem score and 1 woman for answer about contraception use before pregnancy.

c Missing data from 8 women for self-esteem score and 4 women for answer about contraception use before pregnancy.

Most of participants in the three groups referred to have used contraceptive methods at some point in their lives, with no significant difference among them. Comparing Adults and RPTeens, the last ones referred use of injectable methods more often (65.2% vs 19.2%; OR 7.13 [1.93–26.3]), but there was no significant difference about other types of contraceptive methods. In contrast, when asked about the use of contraceptives right before getting pregnant, most 1^st^PTeens and Adults women referred irregular use (82.6% and 73.1%, respectively, vs 27.6% of RPTeens). The RPTeens referred no use of any contraception more frequently than the control groups (51.7% vs 17.3% of 1^st^PTeens and 26.9% of adults). When asked about their intention to become pregnant, primiparous adolescents were more likely to refer unintended pregnancy when compared with the other groups (75% of primiparous adolescents vs 53.3% of non-primiparous adolescents and 33.3% of adult women) (Table 4).

Between adult women and RPTeens, there were also no difference concerning obstetrical issues, such as quality of prenatal care, mode of delivery, maternal pathological conditions or neonatal outcomes, including Apgar scores and birth weight. Additionally, there was no significant difference about tobacco or alcohol use either.

## Discussion

Our study shows some factors associated with repeat pregnancy in adolescents, such as early sexual debut (before 15 years), first delivery before 15 years of age and drugs use. Additionally, having a second delivery significantly increases the risk of educational interruption in comparison with adolescents who had only one delivery.

These results are in concordance with the literature. Many studies confirm the age of first sexual intercourse, whether consented or not, as a risk factor for repeat adolescent pregnancy, as well as for rapid repeat pregnancy (new pregnancy in less than 2 years after the first delivery).^16^ In our study, this was more evident when we compared non-primiparous adolescents with adult women, as 73.3% of adolescents referred first sexual intercourse before the age of 15, whereas only 23.3% of adults related early sexual debut (OR 9.04; CI 2.80–29.1). The difference in sexual behavior between these groups may be due to an easier exposition to sexual contents alongside with lack of parental counseling and guidance.

Usually, rates in girls under 15 years are unstable because these data routinely collected from statistics from around the world. In our study, few participants had given birth before 15 years, but most had sexual debut before the age of 15 and ∼ 26% of them had sexual relations before 13 years.

Socioeconomic factors, such as low education and low family income, are not so clearly defined as a cause or consequence of early pregnancy. There is evidence that adolescent mothers have lower educational achievements, perhaps because they have never had access to schools, or because they are more likely to interrupt school before or during pregnancy or even after delivery, besides, many may never get the chance to return to it. Investments in health and behavioral education, such as effective interventions to prevent the first and repeated adolescent pregnancies, when combined with community and family participation reinforce the importance of contraceptive counseling for avoiding early pregnancies, thus allowing these women to have the education and economic benefits that schools could offer.^2^ ^17^

In this study, modifiable factors, such as school interruption and poor education, were associated with repeat adolescent pregnancy. This shows the need for care practitioners to undertake efforts to stimulate these girls by explaining the possible consequences of school evasion for them and their babies, while also reinforcing the opportunities they could have by taking control of the decision-making process in their lives.

Adolescent mothers are submitted to several difficulties during transition to motherhood. Their adaptations to this new reality get easier when they have a strong family nucleus that is able to provide the support needed. According to our results, although not significantly different, women tended to have better relationship with their mothers than with their fathers or sexual partners. Other studies^18^ ^19^ found an association between strong family support and fewer rates of teen pregnancies, showing that good communication between adolescent girls and their parents (especially their mothers) discouraged early pregnancy and improved the teenage mothers' psychological adjustment.

Self-esteem is a psychological construction defined as an interpretation of self-concept. It comprehends feelings and thoughts about positive and negative values that a woman attributes to herself. Our results showed that adolescents who have repeat unintended pregnancy tend to present lower self-esteem scores more often than their peers. This finding is concordant with a study that appoints low self-esteem as an independent risk for ineffective use of contraceptives and therefore a risk for unwanted pregnancy.^20^

In addition, RPTeens had significantly more drugs use than adults or teens at first pregnancy. About 35% of adolescents smoke during pregnancy and tend to resume smoking habits during the peripartum period, and they are also often more likely to present alcohol and substance abuse.^3^

Despite the high efficacy of long-action reversible contraceptives (LARC), many adolescents chose less effective forms or no postpartum contraception at all. Some studies point that teenagers have the lowest LARC usage rates, ∼ 4%, compared with any others age group.^8^ In our study, most participants had used contraceptive method at least once in life. However, 65% of non-primiparous adolescents referred to be receiving injectable contraceptives before getting pregnant for the second time, mostly with irregular use. As for LARC usage rate, it was more frequent amongst adult women but still much less often than oral contraceptives (3.8% vs 73%). This should be considered when counseling postpartum contraception, as the literature brings evidence of the safety and effectiveness of LARC insertion in postpartum visit, with lower unintended repeat pregnancy rates (18–20%) and longer interval between pregnancies.^9^

This study had limitations because of the short sample, and the fact that some participants did not answer the self-esteem questionnaires fully, which made it difficult to evaluate the psychological impacts related to adolescent pregnancy. The retrospective nature of the questionnaire may lead to inaccuracy of information, especially about previous use of contraceptives. Another limitation was due to the scenario in which the interviewing took place, as many girls were with their partners or family members and this may have influenced their answers concerning psychological issues and family relationship.

This study evaluated psychological factors among teenagers with two or more births in comparison to primiparous adolescents and adults. Our findings value the importance of modifiable risk factors, significantly associated with unintended repeat pregnancy among adolescents. According to this, it is possible to assume repeat pregnancy in adolescents is a changeable reality alongside all economic, educational and psychological benefits of its prevention.

## Conclusion

An association was established between repeat pregnancy during adolescence and lower education, as these adolescents are more likely to interrupt school and present educational underachievement. Additionally, teenagers with repeat pregnancy are more often likely to present history of drugs use and to initiate early sexual activity (before 15 years). According to this, adolescent repeat pregnancy is more often unintended, probably due to absent or ineffective use of contraceptives. Moreover, these girls tend to present lower self-esteem in comparison with first-time adolescent mothers and adult women.
